# Temperature dependence of band gap in MoSe_2_ grown by molecular beam epitaxy

**DOI:** 10.1186/s11671-017-2266-7

**Published:** 2017-08-15

**Authors:** Byoung Ki Choi, Minu Kim, Kwang-Hwan Jung, Jwasoon Kim, Kyu-Sang Yu, Young Jun Chang

**Affiliations:** 1 Department of Physics, University of Seoul, Seoul, 02504 Republic of Korea; 2 Center for Correlated Electron Systems, Institute for Basic Science (IBS), Seoul, 08826 Republic of Korea; 3 Department of Physics and Astronomy, Seoul National University, Seoul, 08826 Republic of Korea; 4 Korea Materials and Analysis Corp, Daejeon, 34028 Republic of Korea

**Keywords:** MoSe_2_, Temperature-dependent band gap, Thermal stability, Molecular beam epitaxy, Spectroscopic ellipsometry, Time of flight medium-energy ion-scattering spectroscopy

## Abstract

We report on a temperature-dependent band gap property of epitaxial MoSe_2_ ultrathin films. We prepare uniform MoSe_2_ films epitaxially grown on graphenized SiC substrates with controlled thicknesses by molecular beam epitaxy. Spectroscopic ellipsometry measurements upon heating sample in ultra-high vacuum showed temperature-dependent optical spectra between room temperature to 850 °C. We observed a gradual energy shift of optical band gap depending on the measurement temperature for different film thicknesses. Fitting with the vibronic model of Huang and Rhys indicates that the constant thermal expansion accounts for the steady decrease of band gap. We also directly probe both optical and stoichiometric changes across the decomposition temperature, which should be useful for developing high-temperature electronic devices and fabrication process with the similar metal chalcogenide films.

**Electronic supplementary material:**

The online version of this article (doi:10.1186/s11671-017-2266-7) contains supplementary material, which is available to authorized users.

## Background

Two-dimensional layered transition metal dichalcogenides (TMDs) have attracted amplified interests due to interesting physical behaviors such as direct-indirect band gap transition, valleytronics, ferroelectric, and charge-density wave [1–7]. Many semiconducting TMDs possess direct band gap at the K point in monolayer (ML), so that the strong excitonic transition [8–17], and the resulting enhancement of optical behavior for optoelectronic device development are exhibited [18–25]. Especially, the direct band gap (1.55 eV) of MoSe_2_ is close to the optimal band gap value of single-junction solar cells and photo-electrochemical devices [26–30]. In addition, variation of band gap via partial oxidation or temperature control provides potential applications involving external control of optical properties in TMDs, such as optoelectronic devices toward wider light spectrum [31, 32]. However, modulation of band gap has been studied so far by monitoring A exciton peaks in 1-ML MoSe_2_ below 420 K [26], and high-temperature stability has not been addressed for any TMD films. This is partly due to difficulty in preparation of single crystalline TMD films with large uniformity.

Growth of TMD film has been rapidly developing to meet the elevated interests for various ways, such as chemical vapor deposition (CVD), pulsed laser deposition, and molecular beam epitaxy (MBE) [5, 33–35]. CVD has been most widely utilized for crystalline films, but it often provides non-uniform films with small crystalline grains. The state-of-the-art metal-organic CVD growth shows uniform films with polycrystalline grains [36]. On the other hand, MBE has been proved to grow epitaxial films with uniformity for various kinds of TMDs. In addition, in situ reflection high-energy electron diffraction (RHEED) monitoring provides precise control of film thicknesses.

In this paper, we report on the high-temperature optical and stoichiometry properties of epitaxial MoSe_2_ ultrathin films grown by MBE. We analyzed temperature dependence of the band gap of the MoSe_2_ ultrathin films with spectroscopic ellipsometry. We also directly measure the decomposition process in terms of surface crystallinity and stoichiometry.

## Methods

Series of MoSe_2_ films were epitaxially grown on graphenized SiC substrates in a home-built MBE system with base pressures of 1 × 10^−10^ Torr. We used 6H-SiC single crystal substrates, supplied by the Crystal Bank at Pusan National University. We prepared bilayer graphene on the 6H-SiC substrates by annealing at 1300 °C for ~5 min, following the reported recipe [1]. On the graphene surface, we grew epitaxial MoSe_2_ films with lattice mismatch of ~0.3%. Molybdenum and selenium were evaporated with e-beam evaporator and effusion cell, respectively. We deposited the films at growth temperature of 250 °C with growth rate of 0.1 ML/min, followed with post-annealing at 600 °C for 30 min [1]. We monitor the film surface with in situ reflection high-energy electron diffraction (RHEED) with high voltage of 18 kV.

Film crystallinity was checked with high-resolution x-ray diffraction (HRXRD, Bruker, D8 Discover). Spectroscopic reflection measurement was performed with two spectroscopic ellipsometries (JA Woollam, V-VASE), one in atmosphere and the other in a separate ultra-high vacuum chamber. Stoichiometry was analyzed by time of flight medium-energy ion-scattering spectroscopy (TOF-MEIS, KMAC, MEIS-K120) with He^+^ ion beam with 100.8 keV. For estimation of sample thickness, we used bulk density values for SiC with 3.21 g/cm^3^ and for MoSe_2_ with 6.98 g/cm^3^.

## Results and discussion

**Fig. 1:**
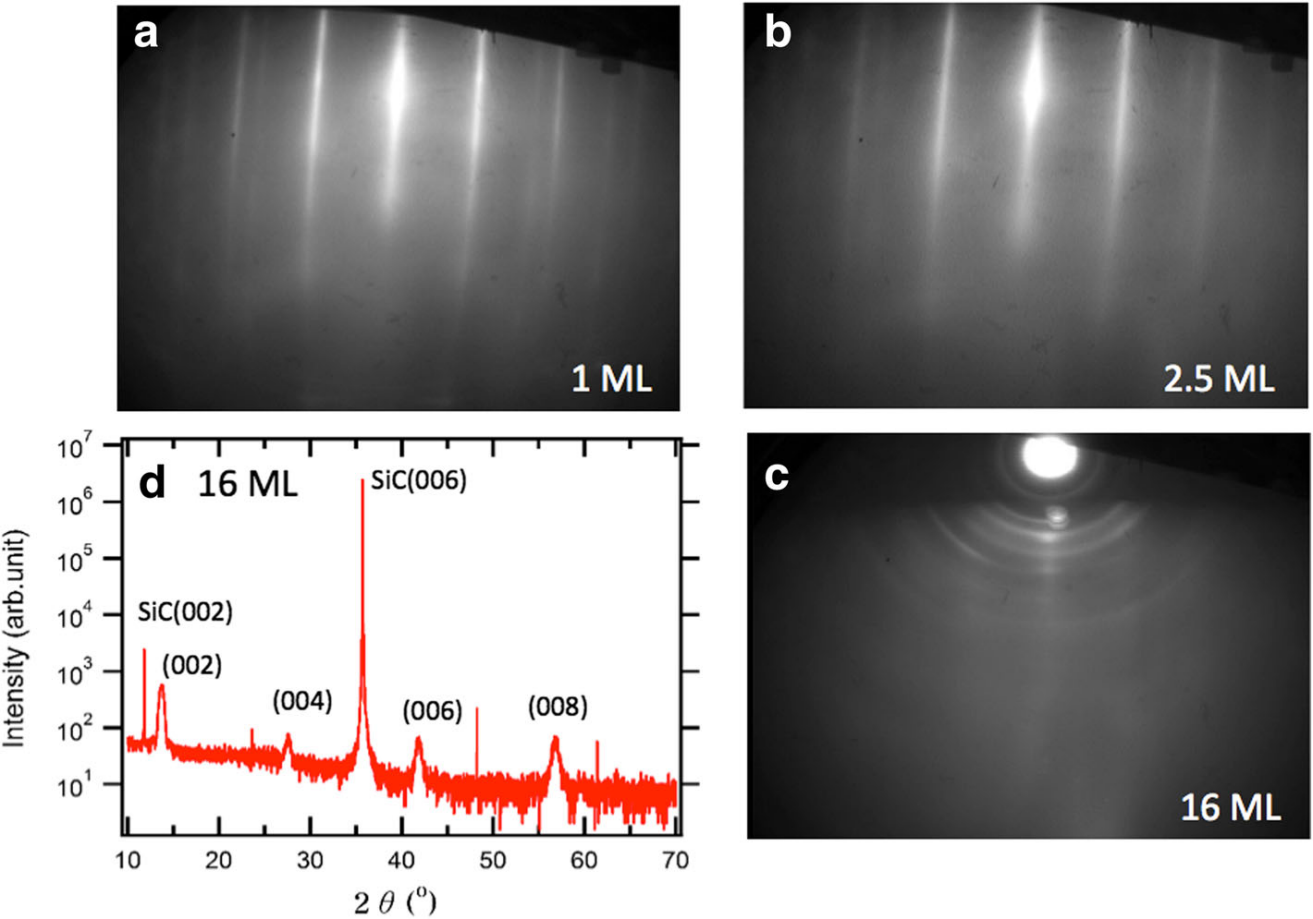
**a–c** RHEED patterns of 1 (**a**), 2.5 (**b**), and 16 ML (**c**) MoSe_2_ thin films on epitaxial graphene are illustrated. **d** XRD data of the 16-ML MoSe_2_ thin film

We fabricated three kinds of epitaxial MoSe_2_ films with different thicknesses (1, 2.5, and 16 ML) on graphene/SiC substrates. In Fig. 1, RHEED images shows epitaxially grown MoSe_2_ films. Well-separated straight lines in Fig. 1a, b indicate electron diffraction from the well-ordered surface crystallinity. Additional lines with different periodicity correspond to the diffraction signal from the underlying graphene probably due to electron penetration through the ultrathin films, which is consistent with the previous reports on MBE-grown MoSe_2_ films [1]. As the film thickness increases, we found a weaker RHEED signal along with rounded spots, which implies in-plane orientation disorder at the surface of the 16-ML film, as shown in Fig. 1c. Figure 1d shows HRXRD pattern of the 16-ML film, which shows only *c*-axis ordered peaks, i.e., (00n), except the very sharp peaks originated from the single crystalline SiC wafer. These *c*-axis diffraction peaks indicate that the 16-ML film possesses periodic layer stacking, even though the top surface may have in-plane disorders. Therefore, we prepared all three epitaxial films with high crystallinity, which are ready for temperature-dependent analysis.

**Fig. 2:**
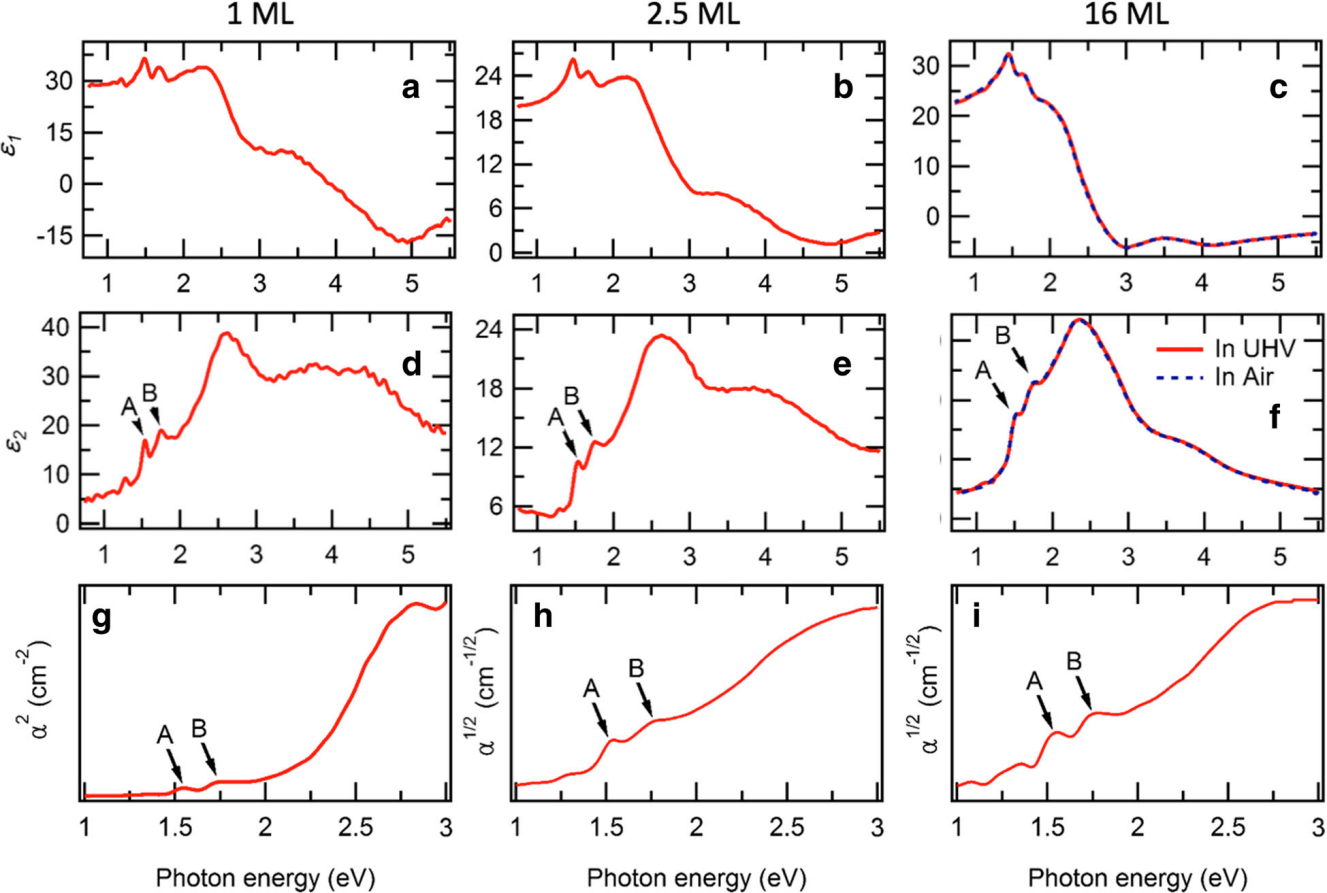
Optical spectra of the 1, 2.5, and 16 ML of MoSe_2_ films. **a**–**c** Real part of the dielectric function (*ε*_1_). **d**–**f** Imaginary part of the dielectric function (*ε*_2_). **g**–**i** Absorption coefficient (*α*) for the case of direct band gap (**g**) and indirect band gap (**h, i**). The peaks labeled *A* and *B* in **d**–**i** correspond to the direct excitonic transition at the K point in momentum space. All the measurements are performed in UHV at room temperature, except the 16-ML MoSe_2_ film measured both in UHV and air

We first obtained room temperature optical spectra of the 16 ML-thick MoSe_2_ both in air and in UHV condition with two distinct ellipsometry spectrometers. As shown in Fig. 2c, f, those two spectra (solid and dashed lines) well overlap and show two characteristic peaks near ~1.5 eV (A) and ~1.7 eV (B). Those two peaks correspond to the two excitonic transitions at the K point of the band structure [37, 38]. Strong spin-orbit coupling induces splitting of degenerated valence band maximum at K point [29, 39–42]. These two exciton peak energies well compared to the reported exciton energy values, ~1.55 and ~1.75 eV, in the exfoliated bulk [38]. Then, we show ellipsometry spectra of the 1- and 2.5-ML samples measured in UHV condition at room temperature, as shown in Fig. 2a–e, respectively. As the film thickness is reduced, the exciton peaks become sharp, probably due to band structure transition from the indirect band gap to the direct one [1, 43]. The ellipsometry spectrum of the 1 ML resembles the reported spectrum of exfoliated 1-ML MoSe_2_ flake [38, 44]. However, the ellipsometry spectrums of few layers-thick MoSe_2_ have yet to be reported. From the ellipsometry spectra, we extracted the two exciton peak energies of all three samples at room temperature. As listed in Table 1, both the A and B exciton peaks show negligible change as the layer thickness decreases, because it relates with the direct band gap, which is insensitive to the thickness-dependent direct-indirect band gap transition. The A exciton band gap (1.54 eV) of the 1-ML sample is close to the values reported in the photoluminescence experiments on mechanically exfoliated [26] and CVD-grown 1-ML MoSe_2_ on SiO_2_ [31, 45], and the ARPES experiments of MBE-grown 1-ML MoSe_2_ on graphene [1].

**Table 1:** Exciton peak energies and the fitting parameters from Eq. (1) for the 1, 2.5, and 16 ML of MoSe_2_ films

Layer thickness (ML)	A exciton (eV, 300 K)	B exciton (eV, 300 K)	*E*_g_(300 K) (eV)	Electron-phonon coupling (*S*)	*E*_g_(0) (eV)	Average phonon energy (meV)
1	1.54	1.75	2.18	3.5	2.32	11.6
2.5	1.53	1.76	1.54	3	1.68	
16	1.54	1.76	1.40	4	1.50	

To extract optical band gap values using Tauc plot, we further converted the ellipsometry spectra into the absorption coefficient *α* of each samples. Since only the 1-ML MoSe_2_ has a direct band gap, we manifest *α* ^2^ and *α* ^1/2^ to estimate the band gap for the 1 ML and the rest of the samples, respectively. As shown in Fig. 2g–i, the absorption spectra also show the two exciton peaks between 1.5–1.75 eV, which is consistent with the reported absorption spectrum of 1-ML MoSe_2_ grown by CVD [44]. In addition to the two exciton peaks, the absorption spectra show a broad peak centered at ~3 eV, corresponding to the charge transfer absorption, and we could extract band gap value using Tauc plot, which is used to determine optical band gap in semiconductors, shown as straight line fittings in Fig. 2g–i. We listed the extracted optical band gap (*E*_g_(300 K)) at room temperature in Table 1, in which the 1-ML value (2.18 eV) is nearly same with the reported band gap measured by scanning tunneling spectroscopy measurements [40]. Contrary to the excition peaks, the optical band gap shows sharp increase when the layer thickness is diminished. Especially, big change of band gap between 1 ML (2.18 eV) and 2.5 ML (1.54 eV) is consistent with the direct-indirect band gap transition in this ML limit [1].

**Fig. 3:**
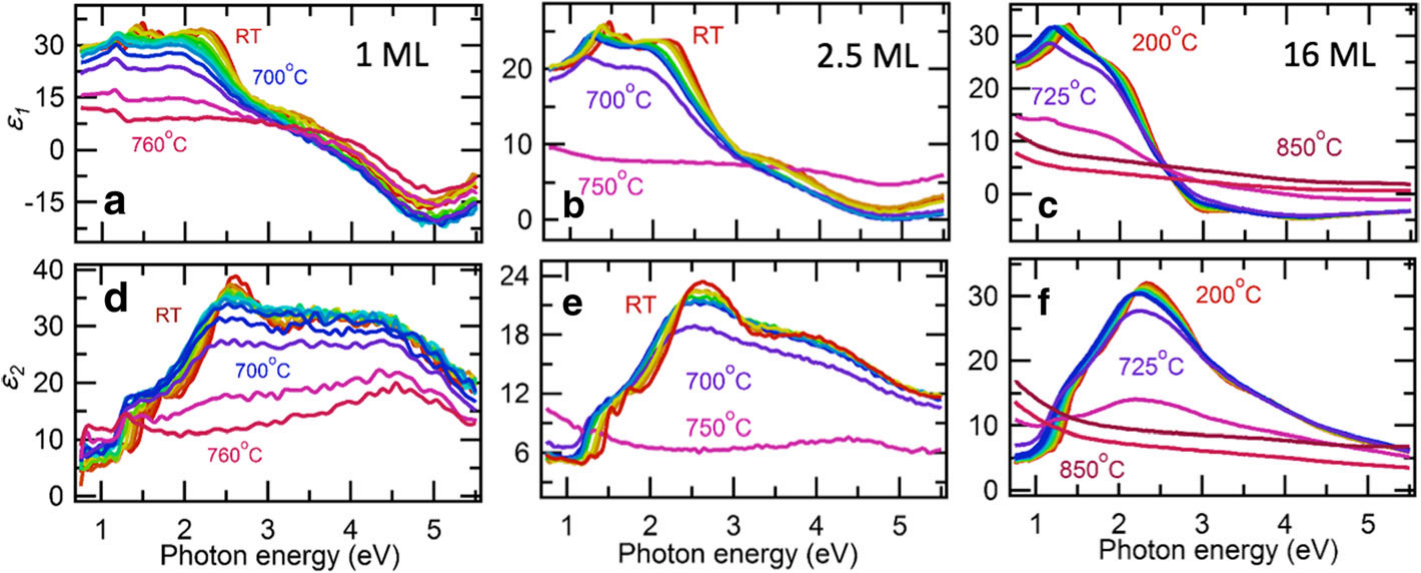
Temperature dependence of optical spectra of the 1, 2.5, and 16 ML of MoSe_2_ films. **a**–**c** Real part of the dielectric function (*ε*_1_). **d**–**f** Imaginary part of the dielectric function (*ε*_2_)

**Fig. 4:**
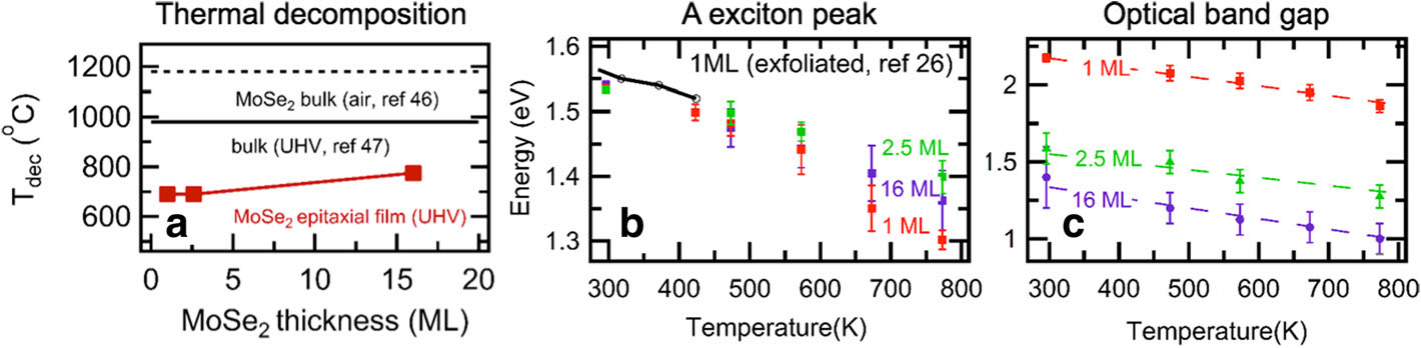
**a** *T*_dec_ of MoSe_2_ bulk and thin films in air or UHV conditions. *Red squares* are from the temperature-dependent optical spectra on the MoSe_2_ epitaxial films, while *black solid* and *dashed lines* correspond to the bulk MoSe_2_ in UHV [47] and air [46] condition in the literatures. **b** Temperature dependence of the A exciton peaks in imaginary part of dielectric functions in Fig. 3d–f. *Black open circles* indicate the A exciton peak values of the exfoliated 1-ML MoSe_2_ taken in the previous report [26]. **c** Temperature dependence of the optical band gap values for the 1, 2.5, and 16 ML of MoSe_2_ films, taken from the absorption spectra

To understand thermal change of the optical band gap, we repeated the ellipsometry measurements while heating the three samples in UHV condition. Figure 3 shows the series of optical spectra for various measurement temperatures ranging from room temperature to 750–850 °C. For every sample, the spectra suddenly lose the characteristic peak structures and become monotonic above different temperatures, which we define as the decomposition temperature (*T*_dec_) for each sample, as we discuss stoichiometry analysis below. *T*_dec_ increases from 700 °C for the 1 ML to 725 °C for the 16 ML. As shown in Fig. 4a, the *T*_dec_ of the ultrathin films in UHV are far lower than those of bulk in air (1200 °C) [46] and in UHV (980 °C) [47]. This implies that the ultrathin MoSe_2_ should be handled for restricted temperature range below the *T*_dec_. When cooled after the thermal annealing cycles below the *T*_dec_, we confirmed restoration of the optical spectra for the 2.5-ML MoSe_2_ (see Additional file 1: Figure S1).

Below the *T*_dec_, we identified gradual red shifts of the most characteristic peaks for all the three samples, as shown in Fig. 3. As shown in Fig. 4b, we extract the band gap values from the A exciton peak positions as a function of measurement temperature (also see Additional file 1: Figure S2). The temperature dependence of the A exciton peak shows nearly linear dependence, which is similar to the one in the exfoliated monolayer for 300–420 K [26]. However, the optical band gap of MoSe_2_ is known to be quite different from the exciton peak due to exceptionally large exciton-binding energy [40].

The linear temperature dependence of the optical band gap over wide temperature range is illustrated in Fig. 4c. Repeating the Tauc plot in Fig. 2g–i, we could extract the optical band gap values from each spectra. All three samples show nearly similar linear dependence of the band gap for the wide temperature range. The linear temperature dependence of the band gap over wide temperature range is similar to one of the other semiconductors [48–51]. We could fit this temperature dependence by using the vibronic model of Huang and Rhys [51, 52];

1 $$ {E}_g(T)={E}_g(0)\hbox{--} S< h\nu >\left[\mathit{\coth}\left(< h\nu >/2{k}_BT\right)\hbox{--} 1\right] $$

where *E*_*g*_(0) is the band gap at 0 K, *S* is a dimensionless electron-phonon coupling parameter, <*hν* > is the average acoustic phonon energy, and the coth term represents the density of phonons at the specific temperature. Shown as dashed lines in Fig. 4c, we could fit the temperature dependence well with *E*_*g*_(0) = 1.5–2.32 eV and *S* = 3–4, while we fixed the value of <*hν* > = 11.6 meV from the previously reported value in the exfoliated monolayer MoSe_2_ [26]. While the fitting parameters are listed in Table 1, the parameters are quite different from the reported values (*E*_*g*_(0) = 1.64 eV and *S* = 1.93) for the exfoliated monolayer MoSe_2_, because they fit the A exciton energy instead of the optical band gap. However, *S* values are quite similar to the reported values for three-dimensional compound semiconductors, such as GaAs and GaP [48]. We note that nearly constant thermal expansion coefficient of MoSe_2_ above 150 K explains the linear reduction of band gap upon heating [53].

**Fig. 5:**
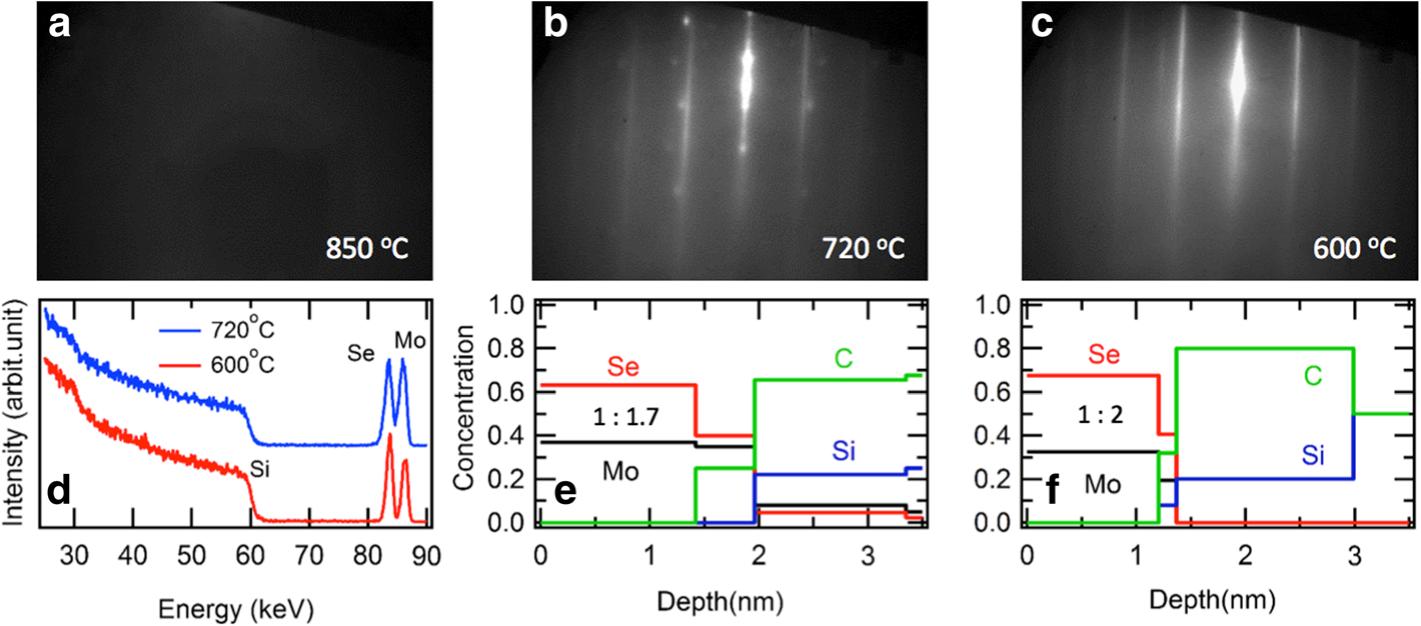
**a–c** RHEED pattern of the 2-ML MoSe_2_ films after annealing at 850 (**a**), 720 (**b**), and 600 °C (**c**) in UHV condition. **d** TOF-MEIS spectra of the 2-ML MoSe_2_ films after annealing at 720 °C (*blue*) and 600 °C (red). **e, f** Depth profile of chemical composition of the annealed films at 720 °C (**e**) and 600 °C (**f**), obtained from the TOF-MEIS analysis. Note that the stoichiometric ratio of Mo:Se is 1:1.7 and 1:2 for the 720 and 600 °C samples, respectively

To understand the abrupt change of the optical spectra above the *T*_dec_ in Fig. 3, we further analyzed surface crystallinity and stoichiometry by utilizing RHEED and TOF-MEIS on the separately prepared 2-ML films, as shown in Fig. 5. The RHEED images show dramatic changes among the samples with different post-annealing temperatures (850, 720, 600 °C) in UHV environment. The sample annealed at 600 °C maintains the similar streaky pattern with the as-grown samples, shown as Fig. 1a, b. However, the 720 °C sample shows additional spots, and the 850 °C sample shows no diffraction signal due to lack of long-range crystalline order. To analyze the amount of decomposition, we performed TOF-MEIS on the 720 and 600 °C samples. The raw spectra in Fig. 5d show similar features except the ratio difference between the Se and Mo peaks between 80 and 90 keV. After modeling with assumption of uniform slab geometry and bulk densities, we obtained depth profile of chemical stoichiometry for both samples. As shown in Fig. 5f, the 600 °C sample shows 1:2 ratio for Mo and Se and the film thickness of ~1.3 nm, indicating preserving the stoichiometry of the as-grown state up to 600 °C. However, the 720 °C sample shows reduced ratio of 1:1.7 and increased thickness of ~1.6 nm, indicating selenium deficiency and surface roughening upon heating across the *T*_dec_. Therefore, the MoSe_2_ layer begins to disorder and decompose at 720 °C, and then remains a disordered molybdenum layer in 850 °C. These direct evidences should be helpful for designing high-temperature fabrication process based on the similar kinds of metal chalcogenide films.

## Conclusions

We prepared a set of MoSe_2_ ultrathin films epitaxially grown by MBE. From the temperature-dependent optical spectra between room temperature to ~850 °C, we identified the thickness-dependent *T*_dec_ and the temperature dependence of band gap. The linear decrease of the band gap is well understood with the vibronic model of Huang and Rhys. Such high-temperature characters should play an important role for development of electronic and optoelectronic devices based on the related metal chalcogenide films.

## Additional file

Additional file 1: Figure S1 a, b. Optical spectra (ε_2_ (a) and ε_2_ (b)) of 2.5-ML MoSe_2_ at 100 °C cooled after thermal annealing at the indicated temperatures. The spectra remain nearly same up to 650, indicating that the observed spectral changes in Fig. 3 are mostly due to the reproducible thermal effect. Figure S2 Expanded plot of temperature dependent optical spectra of the 1, 2.5, and 16 ML of MoSe_2_ films to show detailed thermal shifts; a-c Real part of the dielectric function (ε_1_), d-f Imaginary part of the dielectric function (ε_2_). (DOCX 482 kb)

## Abbreviations

CVD: Chemical vapor deposition;
*E*_g_(0): Band gap at 0 K;
*E*_g_(300 K): Band gap at 300 K;
HRXRD: High-resolution x-ray diffraction;
MBE: Molecular beam epitaxy;
RHEED: Reflection high-energy electron diffraction;
T_dec_: Decomposition temperature;
TMD: Transition metal dichalcogenide;
TOF-MEIS: Time of flight medium-energy ion-scattering spectroscopy;
