# Supplementary material for: Temperature dependence of band gap in MoSe2 grown by molecular beam epitaxy
Source: Nanoscale Res Lett. 2017 Aug 15;12:492. doi: 10.1186/s11671-017-2266-7 (PMC5557720; doi:10.1186/s11671-017-2266-7)

**Suplementary Material for**

Temperature dependence of band gap in MoSe_2_ grown by molecular-beam epitaxy

**Author List**

1. Byoung Ki Choi, Department of Physics, University of Seoul, Seoul 02504, Republic of Korea, bk901225@gmail.com

2. Minu Kim, Center for Correlated Electron Systems, Institute for Basic Science (IBS), Seoul 08826, Republic of Korea & Department of Physics and Astronomy, Seoul National University, Seoul 08826, Republic of Korea, minu.kim84@gmail.com

3. Kwang-Hwan Jung, MEIS Dev. Team, Korea Materials & Analysis Corp., Daejeon 34028, Republic of Korea, kh.jung@kmac.com

4. Jwasoon Kim, MEIS Dev. Team, Korea Materials & Analysis Corp., Daejeon 34028, Republic of Korea, jwas.kim@kmac.com

5. Kyu-Sang Yu, MEIS Dev. Team, Korea Materials & Analysis Corp., Daejeon 34028, Republic of Korea, ksyu@kmac.com

6. Young Jun Chang*, Department of Physics, University of Seoul, Seoul 02504, Republic of Korea, yjchang@uos.ac.kr (Corresponding Author)

**Full Postal Address of the Submitting Author**

Young Jun Chang

163 Siripdaero, Dongdaemun-gu, University of Seoul, Dept. of Physics, Bldg 14-217, Seoul, 02504 Republic of Korea

**Figure captions**

Figure S1. **a, b** Optical spectra (ε_2_ (**a**) and ε_2_ (**b**))of 2.5 ML MoSe_2_ at 100 °C cooled after thermal annealing at the indicated temperatures. The spectra remain nearly same up to 650, indicating that the observed spectral changes in Fig. 3 are mostly due to the reproducible thermal effect.

Figure S2. Expanded plot of temperature dependent optical spectra of the 1, 2.5, and 16 ML of MoSe_2_ films to show detailed thermal shifts; **a**-**c** Real part of the dielectric function (ε_1_), **d**-**f** Imaginary part of the dielectric function (ε_2_).

Figure S1.


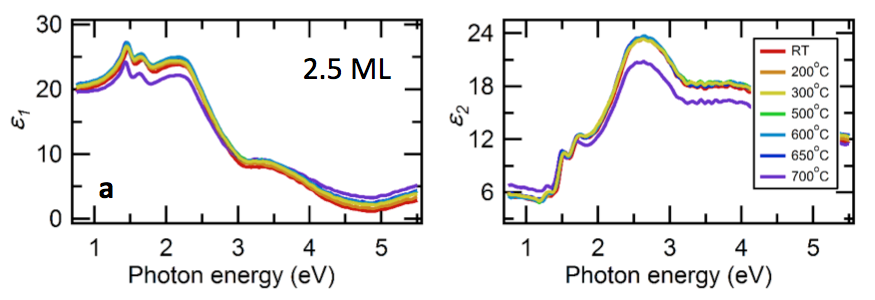


Figure S2.


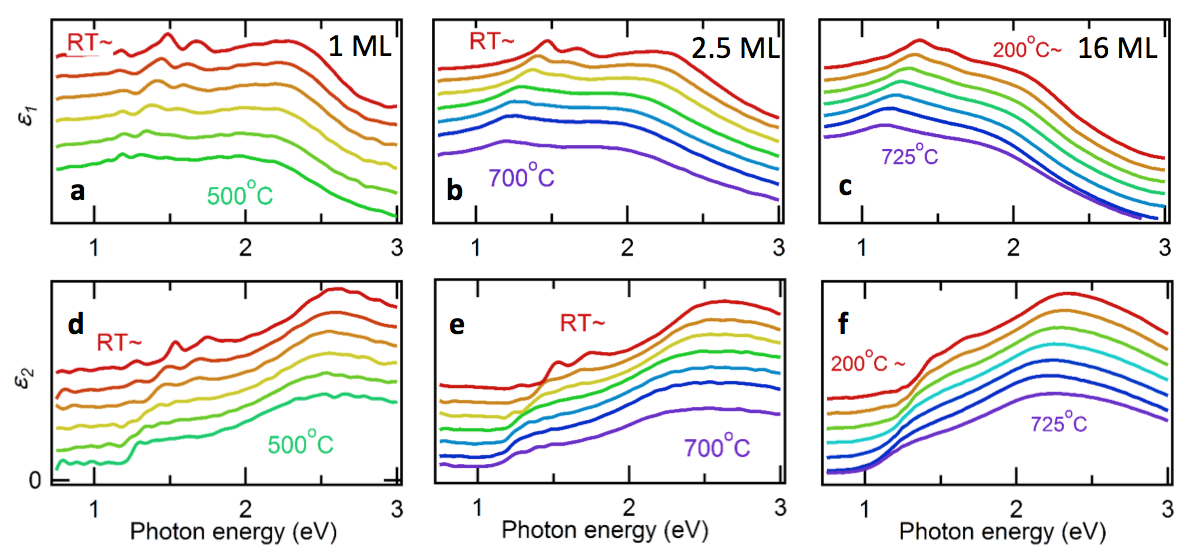

Supplement: Additional file 1: Figure S1 a, b. — Optical spectra (ε2 (a) and ε2 (b)) of 2.5-ML MoSe2 at 100 °C cooled after thermal annealing at the indicated temperatures. The spectra remain nearly same up to 650, indicating that the observed spectral changes in Fig. 3 are mostly due to the reproducible thermal effect. Figure S2 Expanded plot of temperature dependent optical spectra of the 1, 2.5, and 16 ML of MoSe2 films to show detailed thermal shifts; a-c Real part of the dielectric function (ε1), d-f Imaginary part of the dielectric function (ε2). (DOCX 482 kb) [file 11671_2017_2266_MOESM1_ESM.docx]
